# Role of Clinical Pharmacists in Intensive Care Units

**DOI:** 10.7759/cureus.17929

**Published:** 2021-09-13

**Authors:** Enrique Arredondo, George Udeani, Michael Horseman, Trager D Hintze, Salim Surani

**Affiliations:** 1 Pharmacy, Irma Lerma Rangel College of Pharmacy, Texas A&M Health Science Center, Kingsville, USA; 2 Anesthesiology, Mayo Clinic, Rochester, USA; 3 Medicine, Irma Lerma Rangel College of Pharmacy, Texas A&M Health Science Center, Kingsville, USA; 4 Medicine, University of North Texas, Dallas, USA; 5 Internal Medicine, Pulmonary Associates, Corpus Christi, USA; 6 Clinical Medicine, University of Houston, Houston, USA; 7 Medicine, College of Medicine, Texas A&M Health Science Center, Bryan, USA

**Keywords:** drug cost savings, antibiotic stewardship, qa-icu, critical care, clinical pharmacist

## Abstract

The cost of health care has been rising in the United States and globally and will continue to increase. Intensive care unit (ICU) care carries a significant portion of the cost for the hospitals. The Institute of Medicine and subsequent studies have suggested that medication errors account for significant morbidity, mortality, and cost, frequently encountered in the ICU. Over the past three decades, clinical pharmacists have emerged from dispensing medication to getting involved in direct patient care and have become an integral part of the multidisciplinary critical care team. Clinical pharmacists play a significant role in reducing medication errors and costs, medication reconciliation, antibiotic stewardship, and patient and health care provider education. This review will discuss the health care and ICU cost, the evolving role of clinical pharmacists in managing critically ill patients, and their contributions in the ICU to mitigate the risks, improve patient outcomes, and decrease health care costs.

## Introduction and background

The health care cost is rising globally and in the United States. The first intensive care unit (ICU) was established in 1930, and the clinical pharmacy residency service started simultaneously [1,2]. The ICU comprises a significant portion of the health care cost, especially inpatient care. Intensive unit beds comprise almost 5-10% of the hospital bed but account for almost 20-34% of the inpatient health care cost [3]. Between 2000 and 2010, the cost of critical care has increased from $56.6 billion to $108 billion and is on the rise despite a decrease in the number of critical care beds by 17%. In 2010, critical care expenses represented 13.2% of hospital costs and 4.1% of the national health expenditure, and the proportion of critical care medicine cost to the gross domestic product (GDP) increased from 0.54% to 0.72%, representing a $4.7 trillion increase and are expected to rise [4]. The Critical Care Assessment and Improvement Act of 2014 (S.2966) stated that with an aging American population, it has become more critical to have a coordinated effort, which the nation lacks [5]. In addition, there has been a significant challenge with medication errors. In the Institute of Medicine report, "To Err is Human: Building a Safer Health System" [6], they found that unintentional errors led up to 98,000 death per year, and medication errors accounted for 19% of the error with approximately 7,000 deaths [7,8]. A study suggested that errors in hospitalized patient care account for approximately 400,000 death/year [9], making it the third leading cause of death in the United States. The committee on identifying the preventable errors has suggested approximately 1.5 million preventable adverse events/year in the United States, costing approximately 3.5 billion dollars [10]. However, a recent report suggests that the United States spends more than $40 billion each year on patients who have been affected by medication errors and more than $21 billion yearly on preventable medication errors [11,12]. Medication errors issues have ranged from medication labeling, route-specific problems due to challenges with medication formulation design, legibility, and human error, just to name a few [6]. A report by the Institute of Medicine found that 33% of the medication errors were attributed to labeling and packaging issues [10]. These adverse effects can be adverse events or serious errors, which may be preventable. Rothschild et al. found that 45% of the adverse events were preventable and noted that the most serious adverse events occurred during medication ordering or when administering the medication. Most of those errors were slips and misses rather than a deficit in knowledge bases [13]. There has been a growing consensus on the pharmacist's role in reducing medication errors in the ICU. The clinical pharmacist has been involved in various areas of patient management as a part of the critical care team ranging from medication reconciliation, antibiotics usage, presence in code blue (cardiac arrest) situations, venous thromboembolic disease prophylaxis, assistance in compliance with guidelines, curtailing cost, prevention of medication errors, and resident education, to name a few. The IMS Institute for Healthcare Informatics suggested that $200 billion in the excess cause in health care is attributed to improper and unnecessary use of medications. Of those avoidable health care medication costs, almost $106 billion is due to non-adherence to the medication. The other etiology has been a delay in implementing evidence-based practice, antibiotic misuse, medication errors, suboptimal generic use, and mismanaged polypharmacy among the elderly [14]. In patients with chronic diseases such as diabetes and hypertension, a lack of understanding of the long-term effects of the disease process contributed to non-adherence. Patient education provided by pharmacists could lead to increased adherence and decreased long-term comorbidities [15,16]. In this brief review, we will try to address the role of clinical pharmacists in the ICU.

## Review

Clinical pharmacist

The specific requirements vary, but, broadly, the candidate who wants to pursue a career as a clinical pharmacist must complete an undergraduate degree in chemistry, biochemistry, or an allied specialty to obtain a Doctor of Pharmacy degree. This educational experience proves them to be very educated and valuable members of the critical care team. Most of the clinical pharmacists who are practicing as the member of the critical care teams have undergone the critical care pharmacy residency program, which helps them further hone their skills in a critical area with rotations in the medical ICU, surgical ICU, trauma ICU, and cardiovascular and neurology/neurosurgical ICU, to name a few. This added training helps them prepare for their role as an instrumental team member for the ICU team. Critical care pharmacy has evolved over the past three decades; its role has been vaguely defined despite its significant contributions. Recently, the American College of Clinical Pharmacy (ACCP) defined clinical pharmacists as an allied health profession that "work directly with physicians, other health professionals, and patients to ensure that the medications prescribed for patients contribute to the best possible outcomes." In contrast, the abridged definition was "Clinical pharmacy is defined as an area of pharmacy with science and practice of rational medication use" [17,18]. In 2020, the Society of Critical Care Medicine and ACCP defined some of the roles of the clinical pharmacist in the ICU, which includes the role in clinical activities, educational activities, scholarly activities, and administrative activities [19]. These areas entail the performance including medical history, evaluating drug therapy, pharmacokinetic monitoring, and evaluating parenteral nutrition orders, providing educational services to other ICU staff and residents, supervising the handling of investigational drugs, monitoring of adverse drug events (ADEs), and their role in pharmacy and therapeutic committees. MacLaren et al. surveyed 1,220 U.S. institutions and received 401 responses representing 493 ICUs. Of the 493 ICUS, 70.8% of them provided direct patient care by clinical pharmacists, and the educational and administrative services varied [20]. The questions have been raised by clinicians and critics as the role of the clinical pharmacist has been tied closely to their knowledge and skills, as they graduate from the pharmacy school, which does not guarantee high performance as team members [2]. This has resulted in the Critical Care Clinical Pharmacy track and residency program to ensure excellence in this area, but these programs are few, and supply is limited. In 2015, a survey analysis was conducted to determine the number of post-graduate year 2 (PGY-2) critical care pharmacy residencies. There were a recorded 116 PGY2 critical care programs at the time [21].

Cost from medication errors

**Figure 1:**
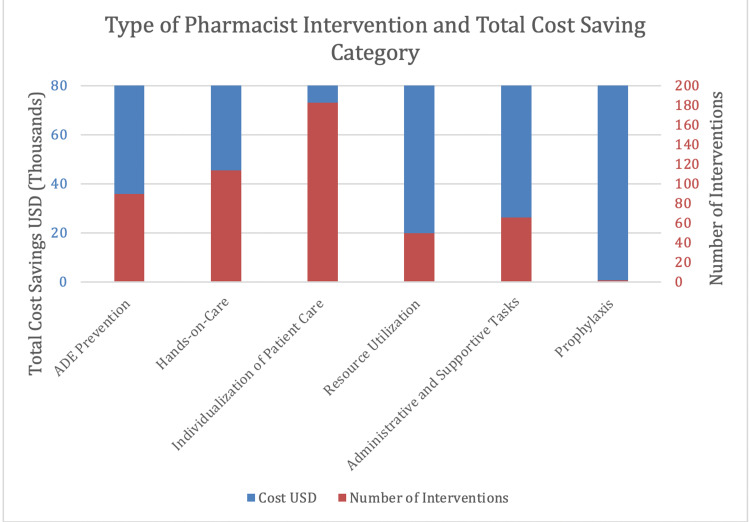
Estimated amount saved based on the number of interventions made in one year.

The estimated cost related to medication errors in the United States has been reported to be $19.5 billion. Of that cost, $17 billion was attributed to additional medical costs as ancillary service, in-hospital and outpatient care, and prescription drug services. Prescribing errors made up to 25% of medication errors. Also, $1.1 million was calculated to be lost workdays based on short-term disability and $1.4 billion due to increase mortality [22,23]. Thomas studied the cost of medical injury in Colorado and Utah and found the total cost of medical errors to be $662 million, and when extrapolated nationally, the cost was found to be $37.6 billion, of which $17 billion were preventable; of that, drug-related errors were listed among the top five errors [24]. The outpatient setting is not immune to medication errors. It has resulted in 1.4 million avoidable office visits, 4 million hospital admissions, and 0.6 million emergency department visits [14]. The physician, nurses, and pharmacist are trained to give error-free care. Despite that, the error does happen. The solutions can be multipronged to prevent errors by improving patient care coordination, engaging the patients, using technology, and having the pharmacist play an integral role by being involved in the whole process including follow-ups. Muñoz-Pichuante and Villa-Zapata analyzed interventions suggested by clinical pharmacists that the providers in the adult ICU accept. In 12 months, 505 interventions were performed in 169 patients, leading to total cost savings of $263,500. Figure 1 illustrates the six different pharmacy services provided and the respective cost savings with the respective number of interventions. Prevention of ADEs provided approximately $88,000 in savings [25].

Cost related to antibiotics resistance

**Figure 2:**
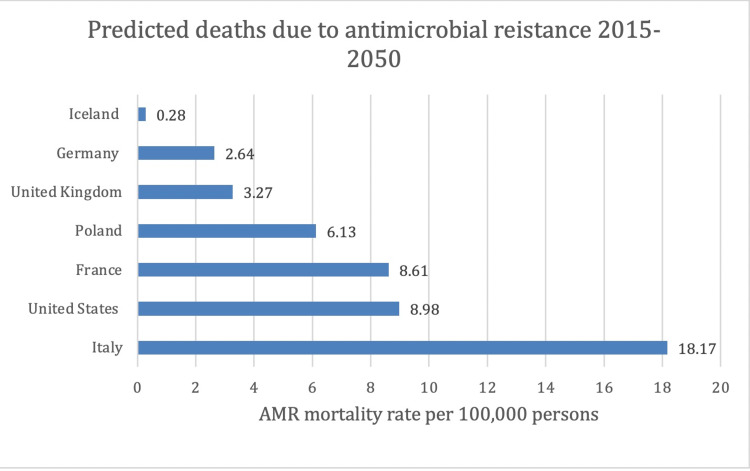
The United States is predicted to be second in AMR mortality per 100,000 persons. AMR, antimicrobial resistance

Cost related to misuse of antibiotics in the United States is estimated to be $20 billion for health care expenses and about $35 billion for loss of productivity [26]. According to the Centers for Disease Control and Prevention (CDC), antibiotics were prescribed for 68% of acute respiratory infections during outpatient visits, and of those, 80% were unnecessary [27]. In addition, CDC released a report in 2017 detailing antibiotic use in the United States and found that the antibiotic prescription per provider rate was 283, and 258.2 million antibiotic prescriptions were provided in 2017 [28]. The inappropriate use of antibiotics has led to the emergence of antibiotic resistance, *Clostridium difficile* infections, and increasing cost. Moreover, prolonged therapy, expensive antibiotics, and excessive use of antibiotics for empirical treatment account for the high cost of antibiotic misuse and cost. These issues are even more challenging in ICUs worldwide. Nosocomial infections have been one of the critical factors leading to high mortality in the ICU. The emergence of resistant gram-positive and gram-negative bacteria has posed a significant challenge in the management of critically ill patients. Several studies have shown a close association between the previous usage of antibiotics and resistance [29-31]. Besides the usage, prolonged hospitalization has also been implicated in predisposing patients to drug-resistant infections [32]. Several strategies have been suggested in preventing antimicrobial resistance (AMR), such as implementation of protocol and guidelines, hospital formulary restrictions, using narrow spectrum and older antibiotics, quantitative cultures and assessment of risk, antibiotic combination therapies, infectious disease specialist consult, antibiotic cycling, area-specific antibiotic therapies, antimicrobial de-escalation strategies, and utilizing clinical pharmacists, to name a few [31]. These strategies where clinical pharmacists assist in minimizing the risk of multidrug-resistant organisms are vital, as demonstrated in Figure 2; the United States is projected to have the second-highest AMR mortality rate per 100,000 persons [33]. The Infectious Disease Society of America has advocated the antimicrobial stewardship (AS) program. Toth et al. studied the implementation of a care bundle for evaluating an AS program. The study involved a group where a stewardship pharmacist was involved in patient care, and the control group had no stewardship pharmacist involved. They found that in the study group, 168 interventions were made, and the acceptance rate was 91%. Moreover, the rate of de-escalation of the antibiotics rose from 72% to 90%. There was an improvement in the quality indicators, which increased from 16% to 43% [34].

A clinical pharmacist is helpful in drug dosing by application of pharmacokinetic and pharmacodynamics concepts. Studies have shown that achieving appropriate pharmacodynamics magnitude can help in achieving clinical success [35,36]. One of the other challenges in the ICU is the clinician being more focused on the clinical aspect of patient care and management. Having a clinical pharmacist on rounds has helped in de-escalating the therapy and achieving the optimal serum concentration [37]. In a position paper by the American Society of Health-System Pharmacists (ASHP), they proposed the pharmacist's role in AS and prevention and control [38]. It emphasizes the pharmacist's role in the area of promoting optimal use of antimicrobial agents by encouraging multidisciplinary collaboration within the health system by being involved in pharmacy and therapeutic committee, working with the microbiology person in the laboratory, conducting and operating AS programs with utilizing the information technology, and facilitating safe medication practices. In addition, pharmacists can be valuable resources in educational activities, especially for nurses and the residents, as well as in patient education regarding drug administration and safety. Pharmacists can also play an integral role in the reduction of infection transmission by their participation in infection control and prevention, use of single-dose packages rather than multi-dose packages or containers, encouraging immunization, advocating adherence to standard precautions for the caregivers, and helping achieve zero tolerance for health care infections [38,39]. In a time when there is a severe shortage of critical care physicians, a high turnover of nursing staff, and a constant flow of new residents in the critical care area, the need and role of clinical pharmacists in the ICU is needed now more than ever. Leache et al. conducted a retrospective observational study evaluating clinical pharmacist interventions' clinical and economic impact on antimicrobials in an ICU. Within the five-month study, 212 drug-related problems were detected in 114 patients, with 18 being medication errors. The interventions resulted in approximately $12,800 in potential savings [40].

Role of clinical pharmacist in the prevention of medication errors

The errors by the health care providers, and specifically the medication errors, have been briefly discussed earlier. The Harvard Medical Practice was one of the first to describe injury related to adverse events [41]. This initial study prompted the other groups to study the incidence and preventability of the adverse events. The Adverse Drug Prevention Group investigators found the rate of ADEs to be 11.5/1,000 patient days and 6.1/100 admissions [42,43]. They also noted that the highest rate of ADE occurred in the medical ICU, which was 19.4/1,000 patient days. The majority of studies have relied on voluntary reporting and chart review, which may underestimate the real issue. In a study by Kopp et al., they found a high incidence of ADE. They found that potential ADE occurred mainly in the dispensing and administration stages. They also noted that all preventable ADEs occurred mainly in prescribing and administration stages, accounting for 77% and 23%, respectively [42]. The computerized physician order entry (CPOE) has emerged to prevent medication error mainly due to legibility issues. On the other hand, CPOE has also emerged as facilitating the errors besides reducing them. This has been felt secondary to fragmented CPOE display, separation of function, facilitating double dosage, and incompatible orders entry [44,45]. Nebeker et al. studied ADEs in a highly computerized hospital. Surprisingly, they found 483 clinically significant ADEs among 937 hospital admissions; in other words, 52/100 admissions were attributed to ADEs. ADEs resulting in serious harm comprised 9%, 22% of ADE required interventions and monitoring, and 32% and 11% required intervention and monitoring alone, respectively. Most ADE was in the ordering stage (61%), and 13% were in the administration stage [46]. The studies have also noted that recognition and documentation of ADE are rare [46,47], so we may be seeing the tip of an iceberg.

 In another study on medication errors in the critical care units, Kopp et al. found that 37% of the ADE were non-preventable. Of the clinically important medication errors, 83% were potential ADEs and 17% were preventable ADEs. They also found that one of the most important causes of medication error was the lack of accurate knowledge by the prescriber [42].

Role of pharmacist in critical care

A patient admitted to the critical care unit is now more complex than ever, presenting with acute illness and several chronic conditions. Mortality rates have decreased as the pharmacy-occupied bed ratio increased; it was felt mainly due to the direct involvement of pharmacists in inpatient care [48,49]. The care has been even more challenging in the phase of medication reconciliation and the drug interaction, especially when patients take several over-the-counter medications before their acute illness. Pharmacist participation in the critical care multidisciplinary rounds has been shown to prevent errors and help reduce the drug cost in the ICU [50-53]. Over the last two decades, the role of the clinical pharmacist has expanded in U.S. hospitals. In a study by MacLaren et al., they surveyed 383 institutions encompassing 1034 ICUs. They found that direct pharmacy activities were provided in approximately 63% of ICUs. The pharmacists were attending the ICU rounds 4.4 ±1.5 days per week. They were involved in patient care for approximately half of their time, and the remaining time was utilized in drug distribution, administration, and educational activities [54]. Another study showed the role of clinical pharmacists in cost savings in a surgical ICU by helping in medication de-escalation, adjustments of medications, non-formulary drug request challenges, and ADE avoidance [55]. Another study randomized the evaluation of pharmacist-initiated intervention in a teaching hospital with 1,200 beds. A total of 5,590 drug profiles were reviewed, with 1,226 interventions identified. Of the interventions, 79% were classified as quality improvement and 21% as cost-saving interventions. Patients who were randomized to the intervention group with a clinical pharmacist had 41% lower drug cost [56]. The pharmacist, besides being part of the critical care multidisciplinary team, has also been involved in drug use evaluation program, cardiopulmonary resuscitation (CPR) team, compliance with venous thromboembolism prophylaxis, drug safety, error prevention, medication counseling, drug information services, clinical research, medication reconciliation, and proper medication history taking. The American College of Critical Care Medicine in their recommendation has stressed the importance of pharmacy services. Pharmacists have also been shown to help achieve clinical endpoint by minimizing fluid intake in patients on fluid restrictions, venous thromboembolism prophylaxis, or drug level monitoring [57,58].

The pharmacist's participation in medical emergencies, such as cardiac arrest situations, has been seen in one-third of U.S. hospitals. The benefit of a pharmacist has been suggested in avoiding ADE in those situations [59]. Another study by the same author showed that pharmacist involvement and participation in the CPR team resulted in a significant decrease in mortality by 19.9 deaths per year per hospital [60]. ASHP supports the participation of pharmacy residents in medical emergencies as a part of training and research. Increasing numbers of hospitals now require the presence of pharmacy residents/pharmacists in medical emergencies [61,62]. In a study by Draper and Eppert, non-compliance with the Advanced Cardiac Life Support guidelines occurred in 58.1% of all documented arrests. They also found that compliance with those guidelines was significantly better when pharmacists were present during the resuscitation efforts. (59.3% vs. 31.9%) [63].

The pharmacist has also helped manage several medications, drug interactions, and compliance with the quality outcome. The study has shown better drug management and cost when pharmacists managed epileptic drugs [59].

Thromboembolic events have led to an increase in ICU admissions. These events have been linked to an increase in ICU length of stay and mortality [64]. In a study of 141,079 patients by MacLaren et al., mortality rates and bleeding complications were higher in the ICUs where thromboembolic or infarction-related events were managed without the clinical pharmacy services. They also found that the ICU length of stay, drug cost, and patient care cost was significantly higher in the ICU where a clinical pharmacist was not involved in direct patient care [65]. The clinical pharmacist's involvement in monitoring the QTc interval prolonging medication also helped reduce the QTc prolongation and helped in improved outcomes. It occurred in 19% of patients compared to 39% of patients where the clinical pharmacy was not involved in QTc interval prolonging medications [66]. The clinical pharmacist has also been of great help as a part of the multidisciplinary critical team in the medication dose adjustment of the critically ill patients receiving continuous renal replacement therapy (CRRT). In a study, almost 91% of the pharmacy dose physicians accepted adjustment recommendations. This resulted in the cost-saving of almost $2,400 per CRRT patient [67].

The challenging area has been medication reconciliation. The medication reconciliation error has been responsible for 40% of medication errors and patient morbidity and mortality [68,69]. Medication reconciliation has been labeled as the basis of good medicines management. In a study conducted in Ireland, the investigators found medication non-reconciliation in 50% of the 1,245 inpatient episodes, which involved 16% of the 9,569 medications. They found the omission of preadmission medication and failure to reconcile new medication as the most frequent errors. In a study presented at the ASHP 2013 meeting, the authors showed that when pharmacists reconcile the medication list, the accuracy improved from 32.3% to 94.2% [70]. The pharmacy role has also been instrumental in managing sedation among critically ill patients on mechanical ventilation in ICU. The study by Forni et al. looked at the impact of tele-ICU on sedation management among the ventilated patients. They found that the addition of tele-pharmacy during the off hours resulted in significant sedation interruptions. They found that having a tele-ICU pharmacist had a beneficial role in sedation management besides other benefits, which a clinical pharmacist may provide in the medication management [71].

Impact of ICU clinical pharmacist in the COVID-19 pandemic

Since the COVID-19 global pandemic, demand for critical care services and health professionals surged drastically. When the United States shut down in March of 2020, the national estimate of ICU beds occupied was 61% [72]. However, as the pandemic progressed, hospitals have been reaching total capacity, leading to an overflow of COVID patients. Typical COVID -19 patients had complex management of medications, intubation, and different pharmacokinetics, with many different types of comorbidities [73,74]. This led to the integration and enhancement of a critical care pharmacist within the health care team to manage the overload of COVID-19 patients in the ICU setting.

Medication errors were at a high potential of occurring due to the high demand of medication administration of COVID-19 patients and the workload of all health care professionals. Before a health crisis like the COVID-19 pandemic, it has been reported that the risk of errors and incidents was estimated to be between 15% and 20% [13]. To mitigate some of these potential errors, critical care pharmacists' roles were collaborating with health care team members to construct institutional protocols. Some of these protocols pertained to COVID-19 intubation, nutrition, heparin dosing, or the use of certain antibiotics [75]. In addition to minimizing drug errors, critical care pharmacists played a critical role in continuously analyzing published data and managing any clinical trials related to any potential COVID-19 treatments [75,76]. A study conducted by Stephen Ward et al. evaluated the impact of involving clinical pharmacists in the rehabilitation and recovery clinics for those patients discharged following COVID-19 related ICU admission. Table 1 demonstrates the number of interventions (N=64) made by a clinical pharmacist for 39 patients following the discharge of the COVID-19 ICU; 65% of the interventions were graded as Eadon 4, meaning significant intervention resulting in improved standards of care [77].

**Table 1:** Number of interventions stratified by Eadon criteria and the estimated cost avoidance in U.S. dollars [77].

	Potentially lethal	Potentially serious	Potentially significant	Minor	Totals
Eadon criteria	6	5	4	1-3	N/A
Number of interventions	0	2	41	21	64
Cost avoidance (U.S. dollars)	0	$3,232.89-$4,914	$5,301.95-$12,296.01	$0-$260.01	$8,534.84-$17,470.01

## Conclusions

In a short span, clinical pharmacists have established their place in direct patient care. The horizons of a clinical pharmacist have broadened significantly over the past decade from the management of pharmacodynamics and pharmacokinetics to being involved as an integral part of the multidisciplinary critical care team. Their role has expanded in drug management, medication reconciliation, involvement in clinical research, and education. In addition, through the COVID-19 pandemic, clinical pharmacists have proven essential health care team members to increase safety and outcomes in the ICU and post-recovery phase. In essence, from being only involved in dispensing medicine three decades ago, they have become an integral part of the health care team, and their roles are expanding. Hospitals that do not have these services should plan to have them added as a part of their armamentarium. The efforts and resources of pharmacy schools, organizations, and hospitals must be directed to train the clinical pharmacists and specialty clinical pharmacists to meet the demand and upcoming challenges.
